# Supplementary material for: Examination of the proximodistal patellar position in small dogs in relation to anatomical features of the distal femur and medial patellar luxation
Source: PLoS One. 2021 May 28;16(5):e0252531. doi: 10.1371/journal.pone.0252531 (PMC8162663; doi:10.1371/journal.pone.0252531)
Supplement: S1 Table — (DOCX) [file pone.0252531.s002.docx]

| **PLL/PL** | | **Coefficient** | **95% CI** | **p** | **Adj. R^2^** |
| --- | --- | --- | --- | --- | --- |
| **Joint angle**  **Cons** |  | -0.00226  2.09 | -0.00549–(-0.000973)  1.80–2.39 | 0.169  <0.001 | 0.0093 |
| **Sex**  **Cons** | Spayed  Male  Female | -0.0238  -0.0542  -0.0365  1.91 | -0.137–0.0891  -0.193–0.0848  -0.173–0.0997  1.83–1.99 | 0.676  0.441  0.596  <0.001 | -0.0242 |
| **Age**  **Cons** |  | -0.000456  1.92 | -0.00138–0.000469  1.84–2.00 | 0.330  <0.001 | -0.0004 |
| **Body weight**  **Cons** |  | 0.0111  1.84 | -0.00283–0.0251  1.76–1.92 | 0.117  <0.001 | 0.0151 |
| **Limb side**  **Cons** | Right | -0.0161  1.90 | -0.108–0.0755  1.84–1.96 | 0.728  <0.001 | -0.0090 |

| **AT angle** | | **Coefficient** | **95% CI** | **p** | **Adj. R^2^** |
| --- | --- | --- | --- | --- | --- |
| **Joint angle**  **Cons** |  | 0.0501  132 | -0.0356–0.136  124–140 | 0.249  <0.001 | 0.0035 |
| **Sex**  **Cons** | Spayed  Male  Female | 1.15  -0.783  -2.12  136 | -1.79–4.09  -4.40–2.84  -5.66–1.43  134–139 | 0.438  0.669  0.239  <0.001 | 0.0053 |
| **Age**  **Cons** |  | -0.00870  137 | -0.0332–0.0158  135–139 | 0.483  <0.001 | -0.0052 |
| **Body weight**  **Cons** |  | 0.330  135 | -0.0373–0.698  133–137 | 0.078  <0.001 | 0.0218 |
| **Limb side**  **Cons** | Right | -0.701  137 | -3.12–1.72  135–138 | 0.567  <0.001 | -0.0069 |

| **FC/PL** | | **Coefficient** | **95% CI** | **p** | **Adj. R^2^** |
| --- | --- | --- | --- | --- | --- |
| **Joint angle**  **Cons** |  | -0.00101  1.01 | -0.00226–0.000243  0.896–1.13 | 0.113  <0.001 | 0.0157 |
| **Sex**  **Cons** | Spayed  Male  Female | -0.0453  -0.0137  0.00500  0.936 | -0.0880–(-0.00252)  -0.0663–0.0389  -0.0466–0.0566  0.906–0.965 | 0.038  0.606  0.848  <0.001 | 0.0270 |
| **Age**  **Cons** |  | -0.000259  0.936 | -0.000616–0.0000986  0.907–0.966 | 0.154  <0.001 | 0.0208 |
| **Body weight**  **Cons** |  | 0.00535  0.894 | -0.0000386–0.0107  0.862–0.925 | 0.052  <0.001 | 0.0286 |
| **Limb side**  **Cons** | Right | -0.00980  0.924 | -0.0454–0.0258  0.900–0.948 | 0.586  <0.001 | -0.0072 |

| **TL/PL** | | **Coefficient** | **95% CI** | **p** | **Adj. R^2^** |
| --- | --- | --- | --- | --- | --- |
| **Joint angle**  **Cons** |  | -0.00310  1.75 | -0.00531–(-0.000898)  1.55–1.95 | 0.006  <0.001 | 0.0649 |
| **Sex**  **Cons** | Spayed  Male  Female | -0.0259  0.0210  -0.0244  1.48 | -0.105–0.0532  -0.0763–0.118  -0.120–0.0710  1.43–1.54 | 0.517  0.670  0.613  <0.001 | -0.0192 |
| **Age**  **Cons** |  | 0.000819  1.42 | 0.000187–0.00145  1.37–1.47 | 0.012  <0.001 | 0.0543 |
| **Body weight**  **Cons** |  | 0.00473  1.45 | -0.00515–0.0146  1.39–1.51 | 0.344  <0.001 | -0.0010 |
| **Limb side**  **Cons** | Right | -0.0365  1.49 | -0.100–0.0275  1.45–1.53 | 0.260  <0.001 | 0.0029 |

| **TL/FC** | | **Coefficient** | **95% CI** | **p** | **Adj. R^2^** |
| --- | --- | --- | --- | --- | --- |
| **Joint angle**  **Cons** |  | -0.00171  1.76 | -0.00430–0.000882  1.53–2.00 | 0.193  <0.001 | 0.0072 |
| **Sex**  **Cons** | Spayed  Male  Female | 0.0476  0.0472  -0.0362  1.59 | -0.0418–0.137  -0.0628–0.157  -0.144–0.0716  1.53–1.65 | 0.293  0.396  0.507  <0.001 | 0.0007 |
| **Age**  **Cons** |  | 0.00130  1.52 | 0.000601–0.00200  1.47–1.58 | <0.001  <0.001 | 0.114 |
| **Body weight**  **Cons** |  | -0.00423  1.63 | -0.0155–0.00707  1.56–1.69 | 0.459  <0.001 | -0.0046 |
| **Limb side**  **Cons** | Right | -0.0216  1.62 | -0.0950–0.0517  1.57–1.67 | 0.560  <0.001 | -0.0068 |

MPL signifies the MPL group compared with the control group.

Abbreviations: Adj., adjusted; AT angle, anatomical trochlear angle; CI, confidence interval; DPP, distal patellar position; FC, craniocaudal size of the femoral condyle; MPL, medial patellar luxation; PL, patellar length; PLL, patellar ligament length; PPP, proximal patellar position; TL, femoral trochlear length
