# Supplementary material for: Examination of the proximodistal patellar position in small dogs in relation to anatomical features of the distal femur and medial patellar luxation
Source: PLoS One. 2021 May 28;16(5):e0252531. doi: 10.1371/journal.pone.0252531 (PMC8162663; doi:10.1371/journal.pone.0252531)
Supplement: S2 Table — (DOCX) [file pone.0252531.s003.docx]

| PPP | VIF |
| --- | --- |
| Joint angle | 1.13 |
| Age | 1.20 |
| PLL/PL | 1.33 |
| AT angle | 1.04 |
| TL/PL | 2.26 |
| TL/FC | 2.04 |
| Mean VIF | 1.50 |

| DPP | VIF |
| --- | --- |
| Joint angle | 1.13 |
| Age | 1.20 |
| PLL/PL | 1.33 |
| AT angle | 1.04 |
| TL/PL | 2.26 |
| TL/FC | 2.04 |
| Mean VIF | 1.50 |

| PPP | VIF |
| --- | --- |
| Joint angle | 1.31 |
| Age | 1.12 |
| MPL | 1.39 |
| Mean VIF | 1.28 |

| DPP | VIF |
| --- | --- |
| Joint angle | 1.31 |
| Age | 1.12 |
| MPL | 1.39 |
| Mean VIF | 1.28 |

| AT angle | VIF |
| --- | --- |
| Body weight | 1.06 |
| MPL | 1.06 |
| Mean VIF | 1.06 |

| FC/PL | VIF |
| --- | --- |
| Age | 1.12 |
| MPL | 1.12 |
| Mean VIF | 1.12 |

| TL/FC | VIF |
| --- | --- |
| Age | 1.12 |
| MPL | 1.12 |
| Mean VIF | 1.12 |
