# Supplementary material for: Examination of the proximodistal patellar position in small dogs in relation to anatomical features of the distal femur and medial patellar luxation
Source: PLoS One. 2021 May 28;16(5):e0252531. doi: 10.1371/journal.pone.0252531 (PMC8162663; doi:10.1371/journal.pone.0252531)
Supplement: S3 Table — (DOCX) [file pone.0252531.s004.docx]

| **PPP** | **Coefficient** | **95% CI** | **p** | **Adj. R^2^** |
| --- | --- | --- | --- | --- |
| **Joint angle**  **MPL**  **Cons** | -0.0116  -0.0179  1.43 | -0.0151–(-0.00819)  -0.114–0.0786  1.13–1.73 | <0.001  0.705  <0.001 | 0.695 |
| **DPP** | **Coefficient** | **95% CI** | **p** | **Adj. R^2^** |
| **Joint angle**  **MPL**  **Cons** | -0.0107  0.0554  2.00 | -0.0136–(-0.00779)  -0.0257–0.137  1.75–2.25 | <0.001  0.171  <0.001 | 0.700 |

MPL signifies the MPL group compared with the control group.

Abbreviations: Adj., adjusted; DPP, distal patellar position; MPL, medial patellar luxation; PPP, proximal patellar position
