# Supplementary material for: Examination of the proximodistal patellar position in small dogs in relation to anatomical features of the distal femur and medial patellar luxation
Source: PLoS One. 2021 May 28;16(5):e0252531. doi: 10.1371/journal.pone.0252531 (PMC8162663; doi:10.1371/journal.pone.0252531)
Supplement: S4 Table — (DOCX) [file pone.0252531.s005.docx]

| **Joint angle** | **SM 1^st^ time** | **SM 2^nd^ time** | **SM 3^rd^ time** | **MS** | **Student** |
| --- | --- | --- | --- | --- | --- |
| **#60 Left** | 94 | 91 | 93 | 92 | 92 |
| **#67 Right** | 91 | 90 | 91 | 92 | 91 |
| **#39 Right** | 92 | 91 | 91 | 89 | 94 |
| **#69 Left** | 93 | 93 | 93 | 95 | 94 |
| **#56 Right** | 84 | 85 | 84 | 84 | 86 |
| **#61 Left** | 94 | 93 | 92 | 96 | 93 |
| **#54 Left** | 68 | 68 | 69 | 68 | 69 |
| **#51 Right** | 104 | 101 | 103 | 100 | 100 |
| **#49 Right** | 102 | 103 | 104 | 101 | 100 |
| **#11 Right** | 89 | 89 | 89 | 89 | 89 |

| **PPP** | **SM 1^st^ time** | **SM 2^nd^ time** | **SM 3^rd^ time** | **MS** | **Student** |
| --- | --- | --- | --- | --- | --- |
| **#60 Left** | 0.17 | 0.15 | 0.23 | 0.20 | 0.21 |
| **#67 Right** | 0.24 | 0.28 | 0.24 | 0.16 | 0.23 |
| **#39 Right** | 0.29 | 0.32 | 0.30 | 0.21 | 0.27 |
| **#69 Left** | 0.50 | 0.50 | 0.52 | 0.50 | 0.52 |
| **#56 Right** | 0.28 | 0.33 | 0.35 | 0.38 | 0.37 |
| **#61 Left** | 0.44 | 0.41 | 0.44 | 0.29 | 0.38 |
| **#54 Left** | 0.73 | 0.77 | 0.74 | 0.81 | 0.73 |
| **#51 Right** | -0.01 | 0.07 | -0.02 | 0.05 | 0.06 |
| **#49 Right** | 0.20 | 0.23 | 0.21 | 0.25 | 0.28 |
| **#11 Right** | 0.24 | 0.34 | 0.33 | 0.27 | 0.31 |

| **DPP** | **SM 1^st^ time** | **SM 2^nd^ time** | **SM 3^rd^ time** | **MS** | **Student** |
| --- | --- | --- | --- | --- | --- |
| **#60 Left** | 0.93 | 0.89 | 0.94 | 0.9 | 0.95 |
| **#67 Right** | 1.06 | 1.07 | 1.07 | 0.97 | 1.04 |
| **#39 Right** | 1.05 | 1.03 | 1.01 | 0.95 | 1.00 |
| **#69 Left** | 1.12 | 1.13 | 1.13 | 1.16 | 1.18 |
| **#56 Right** | 0.99 | 1.14 | 1.16 | 1.19 | 1.20 |
| **#61 Left** | 1.04 | 0.99 | 0.99 | 0.96 | 1.02 |
| **#54 Left** | 1.35 | 1.36 | 1.34 | 1.39 | 1.29 |
| **#51 Right** | 0.65 | 0.71 | 0.67 | 0.68 | 0.70 |
| **#49 Right** | 1.01 | 1.05 | 1.03 | 0.98 | 1.01 |
| **#11 Right** | 0.98 | 0.99 | 1.02 | 0.96 | 0.99 |

| **PLL/PL** | **SM 1^st^ time** | **SM 2^nd^ time** | **SM 3^rd^ time** | **MS** | **Student** |
| --- | --- | --- | --- | --- | --- |
| **#60 Left** | 2.20 | 2.04 | 2.07 | 2.03 | 2.11 |
| **#67 Right** | 1.86 | 1.96 | 1.84 | 1.86 | 1.90 |
| **#39 Right** | 1.73 | 1.83 | 1.80 | 1.71 | 1.79 |
| **#69 Left** | 1.76 | 1.98 | 2.09 | 2.02 | 1.93 |
| **#56 Right** | 1.79 | 1.80 | 1.80 | 1.77 | 1.75 |
| **#61 Left** | 2.15 | 2.23 | 2.20 | 2.19 | 2.23 |
| **#54 Left** | 2.20 | 2.27 | 2.25 | 2.27 | 2.25 |
| **#51 Right** | 2.46 | 2.38 | 2.41 | 2.35 | 2.44 |
| **#49 Right** | 1.86 | 1.86 | 1.82 | 1.98 | 1.85 |
| **#11 Right** | 2.11 | 2.22 | 2.16 | 2.16 | 2.13 |

| **AT angle** | **SM 1^st^ time** | **SM 2^nd^ time** | **SM 3^rd^ time** | **MS** | **Student** |
| --- | --- | --- | --- | --- | --- |
| **#60 Left** | 138 | 132 | 137 | 141 | 138 |
| **#67 Right** | 141 | 142 | 141 | 143 | 139 |
| **#39 Right** | 136 | 135 | 134 | 126 | 132 |
| **#69 Left** | 135 | 136 | 136 | 136 | 136 |
| **#56 Right** | 123 | 127 | 124 | 129 | 128 |
| **#61 Left** | 142 | 141 | 143 | 141 | 140 |
| **#54 Left** | 145 | 147 | 146 | 147 | 147 |
| **#51 Right** | 139 | 142 | 140 | 140 | 143 |
| **#49 Right** | 141 | 143 | 141 | 141 | 144 |
| **#11 Right** | 129 | 136 | 135 | 133 | 133 |

| **FC/PL** | **SM 1^st^ time** | **SM 2^nd^ time** | **SM 3^rd^ time** | **MS** | **Student** |
| --- | --- | --- | --- | --- | --- |
| **#60 Left** | 1.03 | 0.98 | 1.02 | 1.06 | 0.97 |
| **#67 Right** | 0.90 | 0.93 | 0.91 | 0.90 | 0.92 |
| **#39 Right** | 0.90 | 0.90 | 0.88 | 0.91 | 0.86 |
| **#69 Left** | 0.89 | 0.99 | 0.98 | 0.95 | 0.94 |
| **#56 Right** | 0.99 | 0.89 | 0.89 | 0.96 | 0.86 |
| **#61 Left** | 0.99 | 1.06 | 1.06 | 0.98 | 1.06 |
| **#54 Left** | 1.13 | 1.15 | 1.18 | 1.15 | 1.18 |
| **#51 Right** | 0.84 | 0.86 | 0.90 | 0.81 | 0.89 |
| **#49 Right** | 1.00 | 0.92 | 0.96 | 0.98 | 0.95 |
| **#11 Right** | 0.98 | 1.04 | 1.01 | 1.02 | 1.01 |

| **TL/PL** | **SM 1^st^ time** | **SM 2^nd^ time** | **SM 3^rd^ time** | **MS** | **Student** |
| --- | --- | --- | --- | --- | --- |
| **#60 Left** | 1.41 | 1.35 | 1.41 | 1.43 | 1.34 |
| **#67 Right** | 1.21 | 1.28 | 1.19 | 1.25 | 1.23 |
| **#39 Right** | 1.36 | 1.49 | 1.44 | 1.34 | 1.35 |
| **#69 Left** | 1.49 | 1.59 | 1.63 | 1.50 | 1.47 |
| **#56 Right** | 1.46 | 1.23 | 1.24 | 1.22 | 1.20 |
| **#61 Left** | 1.71 | 1.71 | 1.80 | 1.51 | 1.57 |
| **#54 Left** | 1.55 | 1.56 | 1.59 | 1.53 | 1.64 |
| **#51 Right** | 1.43 | 1.55 | 1.46 | 1.53 | 1.59 |
| **#49 Right** | 1.29 | 1.21 | 1.22 | 1.37 | 1.36 |
| **#11 Right** | 1.38 | 1.60 | 1.51 | 1.47 | 1.47 |

| **TL/FC** | **SM 1^st^ time** | **SM 2^nd^ time** | **SM 3^rd^ time** | **MS** | **Student** |
| --- | --- | --- | --- | --- | --- |
| **#60 Left** | 1.37 | 1.37 | 1.38 | 1.37 | 1.34 |
| **#67 Right** | 1.35 | 1.38 | 1.32 | 1.34 | 1.39 |
| **#39 Right** | 1.50 | 1.65 | 1.63 | 1.57 | 1.48 |
| **#69 Left** | 1.67 | 1.60 | 1.67 | 1.57 | 1.58 |
| **#56 Right** | 1.48 | 1.37 | 1.39 | 1.40 | 1.27 |
| **#61 Left** | 1.72 | 1.61 | 1.70 | 1.48 | 1.54 |
| **#54 Left** | 1.37 | 1.36 | 1.35 | 1.39 | 1.33 |
| **#51 Right** | 1.70 | 1.80 | 1.61 | 1.78 | 1.88 |
| **#49 Right** | 1.29 | 1.32 | 1.27 | 1.44 | 1.39 |
| **#11 Right** | 1.41 | 1.54 | 1.49 | 1.45 | 1.45 |
