# Supplementary material for: Examination of the proximodistal patellar position in small dogs in relation to anatomical features of the distal femur and medial patellar luxation
Source: PLoS One. 2021 May 28;16(5):e0252531. doi: 10.1371/journal.pone.0252531 (PMC8162663; doi:10.1371/journal.pone.0252531)
Supplement: Attachment — Submitted filename: Dear.docx [file pone.0252531.s006.docx]

Dear. Editor

I think that this manuscript can be rejected.

The authors describe “ Examination of the proximodistal patellar position in small dogs in relation to anatomical features of the distal femur and medial patellar luxation”. This paper is well organized, and I evaluated the author’s efforts highly, Although this study was revealed the proximodistal patellar position. And I think that it is hard to have scientific merits because this manuscript have gross methodological errors.

Thanks.

sincerely yours.
